# Supplementary material for: PEG-Mediated Protoplast Transformation of Penicillium sclerotiorum (scaumcx01): Metabolomic Shifts and Root Colonization Dynamics
Source: J Fungi (Basel). 2025 May 17;11(5):386. doi: 10.3390/jof11050386 (PMC12113252; doi:10.3390/jof11050386)
Supplement: Supplementary file 1 [file jof-11-00386-s001.zip › jof-3607709-supplementary.pdf]

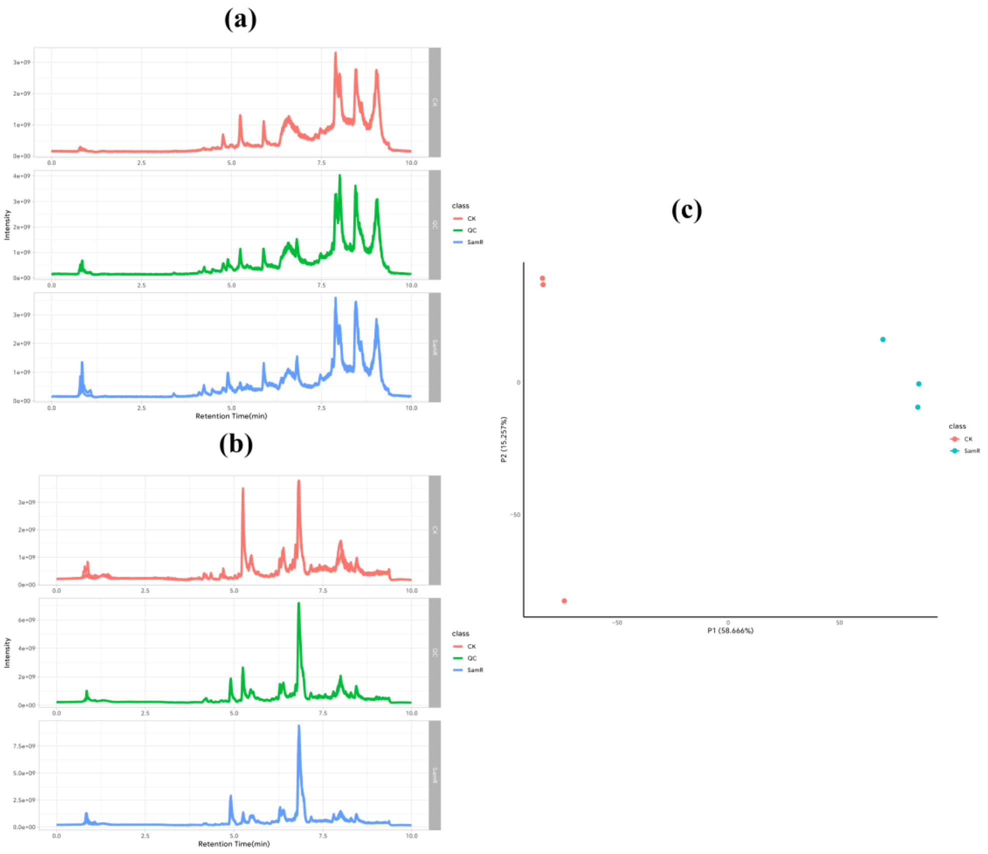

Figure S1. (a-b) Total ion chromatogram (TIC) analysis of fungal metabolites comparing the wild-type strain with the *GFP*-tagged *P. sclerotiorum*-scaumcx01; **(c)** Principal Component Analysis (PCA) illustrating the metabolic differences between the wild-type and *GFP*-tagged strains scaumcx01.

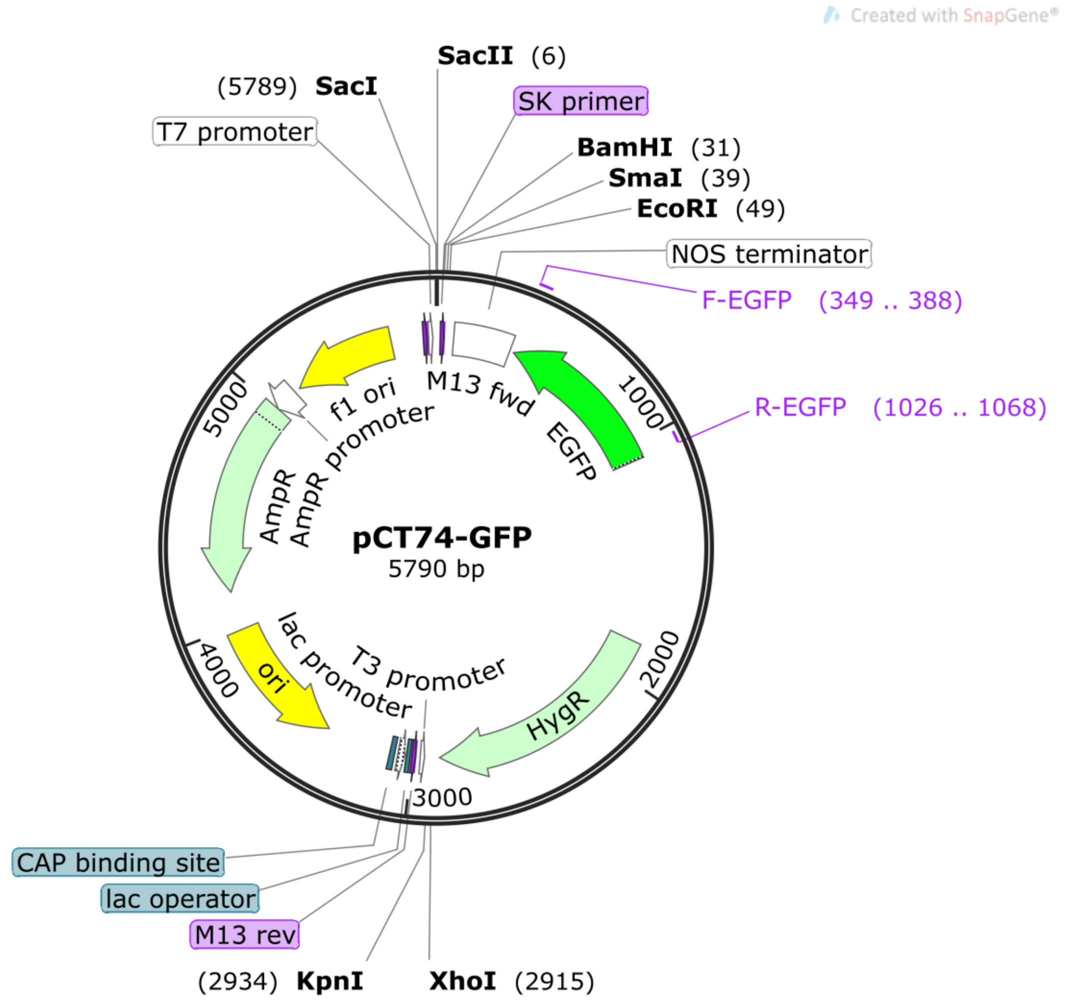

Figure S2. Schematic representation of the pCT74-*GFP* plasmid.

Table S1. Vendor Information and Catalog Numbers for Protoplasting Enzymes.

Enzyme	Vendor	Catalog Number	Activity
Lysing Enzyme	Nanjing Dulai Biology	-	Activity: ≥200 U/mg
Yatalase (Protoplasting Enzyme)	Takara Bio	T017	Chitinase activity: ≥50 U/g powder; Chitobiase activity: ≥500 U/g powder; Cell wall lysis activity: ≈10,000 U/g powder
Cellulase	Feijing Bio	PH9018	Activity: ≥400 U/mg for protein
Pectinase	Feijing Bio	PH1561	Activity: ≥500 U/mg
